# Remote Consultations Versus Standard Face-to-Face Appointments for Liver Transplant Patients in Routine Hospital Care: Feasibility Randomized Controlled Trial of myVideoClinic

**DOI:** 10.2196/19232

**Published:** 2021-09-17

**Authors:** Sarah Damery, Janet Jones, Elaine O'Connell Francischetto, Kate Jolly, Richard Lilford, James Ferguson

**Affiliations:** 1 Institute of Applied Health Research Birmingham United Kingdom; 2 National Institute for Health Research Birmingham Biomedical Research Centre University of Birmingham Birmingham United Kingdom

**Keywords:** digital health, remote consultation, patient satisfaction, feasibility, VSQ-9, secondary care, liver transplant patients, mobile phone

## Abstract

**Background:**

Using technology to reduce the pressure on the National Health Service (NHS) in England and Wales is a key government target, and the NHS Long-Term Plan outlines a strategy for digitally enabled outpatient care to become mainstream by 2024. In 2020, the COVID-19 response saw the widespread introduction of remote consultations for patient follow-up, regardless of individual preferences. Despite this rapid change, there may be enduring barriers to the effective implementation of remote appointments into routine practice once the unique drivers for change during the COVID-19 pandemic no longer apply, to which pre-COVID implementation studies can offer important insights.

**Objective:**

This study aims to evaluate the feasibility of using real-time remote consultations between patients and secondary care physicians for routine patient follow-up at a large hospital in the United Kingdom and to assess whether patient satisfaction differs between intervention and usual care patients.

**Methods:**

Clinically stable liver transplant patients were randomized to real-time remote consultations in which their hospital physician used secure videoconferencing software (intervention) or standard face-to-face appointments (usual care). Participants were asked to complete postappointment questionnaires over 12 months. Data were analyzed on an intention-to-treat basis. The primary outcome was the difference in scores between baseline and study end by patient group for the three domains of patient satisfaction (assessed using the Visit-Specific Satisfaction Instrument). An embedded qualitative process evaluation used interviews to assess patient and staff experiences.

**Results:**

Of the 54 patients who were randomized, 29 (54%) received remote consultations, and 25 (46%) received usual care (recruitment rate: 54/203, 26.6%). The crossover between study arms was high (13/29, 45%). A total of 129 appointments were completed, with 63.6% (82/129) of the questionnaires being returned. Patient satisfaction at 12 months increased in both the intervention (25 points) and usual care (14 points) groups. The within-group analysis showed that the increases were significant for both intervention (*P*<.001) and usual care (*P*=.02) patients; however, the between-group difference was not significant after controlling for baseline scores (*P*=.10). The qualitative process evaluation showed that—according to patients—remote consultations saved time and money, were less burdensome, and caused fewer negative impacts on health. Technical problems with the software were common, and only 17% (5/29) of patients received all appointments over video. Both consultants and patients saw remote consultations as positive and beneficial.

**Conclusions:**

Using technology to conduct routine follow-up appointments remotely may ease some of the resource and infrastructure challenges faced by the UK NHS and free up clinic space for patients who must be seen face-to-face. Our findings regarding the advantages and challenges of using remote consultations for routine follow-ups of liver transplant patients have important implications for service organization and delivery in the postpandemic NHS.

**Trial Registration:**

ISRCTN Registry 14093266; https://www.isrctn.com/ISRCTN14093266

**International Registered Report Identifier (IRRID):**

RR2-10.1186/s13063-018-2953-4

## Introduction

### Background

Increasing the use of technology to reduce pressure on services across the National Health Service (NHS) in England and Wales is a key government target [1]. The NHS Long-Term Plan sets out a strategy for digitally enabled outpatient care to become mainstream across the NHS by 2024 [2]. Central to this strategy is the use of remote consultations, which allows real-time clinician-patient interactions at a distance using video- or telephone-based technology [3,4]. Consequently, there is a growing evidence base assessing the effectiveness of remote consultation in a range of clinical specialties using key metrics such as patient and staff perspectives [4,5], patient acceptability and satisfaction [6,7], health care resource use [8], health outcomes [9], and costs [3].

Despite much of the evidence for the impact of digital technologies on care pathways and service delivery coming from primary care [9-11], studies undertaken in secondary care have found a range of benefits to remote consultations in routine follow-up care. Patients frequently cite advantages relating to personal convenience, such as reduced costs, fewer travel issues, and minimal time off work [3]. For NHS services, there is evidence of a range of incremental benefits, including the efficient use of staff time and physical infrastructure [5]. However, despite the potential benefits of remote consultations in providing follow-up care, uptake in clinical practice remains low [12], and evidence for its impact on health inequalities is equivocal [13]. For many patients, acceptability is mediated by perceived usefulness and security [14], confidence in using the relevant technology [15], and the strength of the patient-clinician relationship [8]. Similarly, although good patient satisfaction can be achieved when remote consultations are used, clinician satisfaction may be reduced [9], particularly when technical issues such as poor audiovisual quality impair patient-clinician communication [3].

Practical implementation issues are often explored superficially in existing studies, and emerging evidence suggests that complexities exist in embedding digital technologies into routine care because of disruption to routines in traditional clinics [16]. Since the emergence of COVID-19, there has been a widespread switch to remote consulting (telephone and on the web) to provide clinical services while mitigating disease risk among vulnerable patients [17-19]. In accelerating the use of remote consultations rapidly, patients and health care professionals have been forced to undergo changes in health care delivery regardless of preference. It is likely that the wholesale shift to remote consultations during COVID-19 has been readily accepted by patients and health care professionals as it was perceived as a temporary measure, with the expectation that follow-up care would revert to prepandemic delivery afterward. A case study of the rapid implementation of virtual consultations for outpatient appointments in orthopedics during COVID-19 showed high levels of satisfaction with appointments but low preferences for remote consultation under *normal* circumstances in which personal safety or easing pressure on services were not the primary drivers of uptake [20]. This suggests that there may be substantial value in assessing the feasibility of implementing remote consultations for follow-up appointments outside of the COVID-19 situation, and an awareness of the enduring perceived advantages and pitfalls of remote appointments may be important when planning how to embed remote consultation within routine practice. This study reports our experiences with the implementation of an outpatient video consultation system before COVID-19.

### Aim and Objectives

The aim of this study is to evaluate the feasibility of using real-time remote consultations between clinically stable liver transplant patients and their hospital physicians using secure videoconferencing technology for routine follow-up appointments and assess whether patient satisfaction differed for intervention patients and those receiving usual care (face-to-face consultations). The study objectives are to (1) assess the rates of recruitment, retention, and crossover between arms; (2) assess the appointment numbers and the technical performance of the remote consultation software; (3) assess patients’ ability to complete clinical testing locally; (4) explore patient satisfaction across key domains of the RAND Visit-Specific Questionnaire-9 (VSQ-9) instrument [21]; (5) monitor questionnaire return rates and data completeness; and (6) assess the feasibility of collecting patient-reported data on health service use, health-related quality of life (HRQoL), and costs. An embedded qualitative process evaluation explored patient and staff experiences.

## Methods

### Design and Setting

A two-armed, parallel group, statistician-blinded feasibility randomized controlled trial (RCT) of the provision of real-time remote appointments via videoconferencing software compared with standard face-to-face consultations (usual care) for delivering routine follow-up to clinically stable liver transplant patients was set up. Participants were recruited from 4 outpatient liver clinics at the Queen Elizabeth Hospital Birmingham (QEHB): primary sclerosing cholangitis, primary biliary cholangitis, alcohol-related liver disease, and autoimmune hepatitis. The trial was registered with the International Standard RCT Number Registry (trial number: 14093266).

The published study protocol [22] outlined a definitive trial to evaluate the effectiveness of remote consultations, with a recruitment target of 180 patients (90 in each arm) that would provide sufficient power to detect a statistically (and clinically) significant difference among groups in the primary outcome measure. However, once underway, poor recruitment meant that a definitive trial would not be possible, and ethical approval was obtained to formally modify the study design to a feasibility RCT. Rather than aiming to provide a definitive assessment of intervention effectiveness, the feasibility RCT reported here placed emphasis on evaluating recruitment and retention, crossover, the feasibility of administering the intervention and collecting outcome data, and an embedded process evaluation. This paper follows the CONSORT (Consolidated Standards of Reporting Trials) extension for randomized pilot or feasibility trials [23], the CONSORT eHealth checklist v1.6 (Multimedia Appendix 1) [24], and the COREQ (Consolidated Criteria for Reporting Qualitative Studies; Multimedia Appendix 2) [25].

### Participants

Clinically stable adult patients (aged ≥18 years) who received a liver transplant 1 to 5 years before baseline were eligible for the study if they (1) could access the myhealth@QEHB patient portal [26]; (2) could arrange for local clinical testing (blood tests, weight, and blood pressure) via their general practitioner (GP) or a dialysis center; (3) had an internet-enabled computer with a camera, running an operating system compatible with the remote consultation software; (4) had follow-up appointments every 3 or 6 months; and (5) could give informed consent. Patients who were unable to speak or read English or those who were involved in another research study involving ongoing questionnaire completion were excluded. Before each clinic, hospital staff screened the day’s appointment list to identify potentially eligible patients according to the time elapsed since transplant. Clinical stability was assessed by the consultant using their judgment of the patient’s liver function, adherence to immunosuppressant medication, and blood test results. Clinically eligible patients were introduced to the study during their appointment by their consultant, who assessed their eligibility further against the other inclusion criteria. Patients interested in participating gave written consent to a member of the research team after their appointment and completed a baseline questionnaire before randomization. Patients who chose not to participate had their reason or reasons recorded.

Participants were randomized in equal numbers to the intervention (myVideoClinic) or usual care (standard face-to-face consultations) arm of the study using the GraphPad web-based randomization tool [27]. Intervention patients were registered on the myVideoClinic system and given instructions to access web-based software training. All patients were registered on the myhealth@QEHB system [26] for the administration of follow-up questionnaires. myhealth@QEHB is a patient records portal developed by the Trust Informatics department, which is currently used by approximately 10,000 patients across 40 clinical specialties. It allows patients remote access to clinical information, including letters, laboratory results, and referrals. Patients can also view appointments, upload or share files on the system, and interact with other patients to create peer-support networks.

### Intervention

Intervention patients received appointment details through post and standard text appointment reminders. Patients were required to undergo clinical tests locally and make results available through myhealth@QEHB [26] before their appointment. For their appointment, patients logged in to myhealth@QEHB to speak to their consultant using an embedded secure videoconferencing platform provided by Vidyo. The Vidyo platform has been used for remote consultations by many health care providers and is underpinned by a comprehensive information governance policy that protects the confidentiality and security of end users according to the national and international governance and data protection standards [28]. Patients could submit 3 questions before their appointment, and consultation audio recordings were available afterward through the myhealth portal. When technical issues occurred during a consultation, the consultant telephoned the patient to finish the appointment and scheduled a face-to-face consultation if necessary.

Patients allocated to the intervention were made aware that it was the option of having a remote consultation and that they could ask for a standard appointment if they wished while still having subsequent appointments remotely. Clinical staff could assign intervention patients to standard appointments at the patient’s request, if there was a clinical need, or if the consultant had not seen preappointment tests for two successive consultations. The statistician, analyzing the primary outcome data, was blinded to participants’ group allocation.

### Usual Care

Patients received standard face-to-face care at the hospital, standard letters notifying them of their appointments, and routine text reminders. They completed clinical tests at the hospital on the day of their appointment, which the consultant reviewed afterward.

### Qualitative Evaluation

An embedded qualitative process evaluation used semistructured interviews to explore participants’ experiences of remote consultations (patients randomized to the intervention and staff administering the intervention). Interviews with staff and patients were conducted using topic guides (Multimedia Appendix 3). These were piloted with members of the study steering group, including the study’s patient representative. Following their 12-month appointment, patients in the intervention arm were contacted by telephone by the research team to ask whether they were willing to take part in an interview. Patients were purposively sampled to ensure diversity in age and sex. At the end of the study, purposively sampled staff (hospital consultants, staff booking appointments, and information technology [IT] support personnel) were invited to take part in an interview. All participants provided written informed consent. Interviews were conducted by 2 experienced female research fellows (JJ, who was qualified at the PhD level, and EOF, who was qualified at the master’s level). Neither interviewer knew the patient participants before the study. Field notes were made by the researcher after each interview. Interviews were audio-recorded, transcribed verbatim, and checked against the recordings for accuracy. Participants were interviewed only once, and they did not have the opportunity to review their transcripts or provide feedback on the findings.

### Outcome Measures

The outcome measures and data collection are summarized in Table 1. The primary outcome was the combined satisfaction score for the three domains of VSQ-9 (convenience of location, getting through to the office by phone, and length of waiting time). Participants rated their satisfaction on a 5-point scale (poor, fair, good, very good, and excellent), which was transformed into a 0 to 100 linear scale, with higher scores denoting greater satisfaction. A 10-point difference between the groups at 12 months was considered clinically significant [29]. Secondary outcomes included recruitment, retention and crossover rates, questionnaire completion rates and return format, system performance, health service use, feasibility of obtaining clinical tests locally, clinical contacts, satisfaction in the other six VSQ-9 domains, and the feasibility of collecting data on patient costs and HRQoL (using EQ-5D-5L) [30].

**Table 1:** Outcome measures and data collection.

Outcome measure		Data collection instrument	Schedule or format^a^
**Primary outcome**			
	(Change in) satisfaction in the VSQ-9^b^ domains of *convenience of location*, *getting through to the office by phone*, and *length of time waiting* [21]	VSQ-9 patient questionnaire	Baseline and 3, 6, 9, and 12 months
**Secondary outcomes**			
	Participant recruitment, retention, and crossover between arms	Routinely collected data	Throughout
	Clinical contacts and nonattendance	Number of appointments	Case report form (consultant completed)
	myVideoClinic system performance	Failed appointments and telephone consultations	Routinely collected metrics
	Patient completion of clinical tests locally	Blood tests, blood pressure, and weight	Case report form (consultant completed)
	Patient satisfaction in the other six VSQ-9 domains	VSQ-9 and patient questionnaire	Baseline and 3, 6, 9, and 12 months
	Questionnaire completion rates	Number of questionnaires completed and mode of return	Baseline and 3, 6, 9, and 12 months
	Feasibility of collecting patient-reported health service use data	Patient questionnaire	Baseline and 3, 6, 9, and 12 months
	Feasibility of collecting patient-reported HRQoL^c^ data	EQ-5D-5L [20] and patient questionnaire	Baseline and 3, 6, 9, and 12 months
	Feasibility of collecting patient-reported cost data (appointment and clinical testing)	Patient questionnaire	Baseline and 3, 6, 9, and 12 months
	Patient and staff experiences of virtual clinics	Semistructured interviews	Semistructured interviews

^a^Data at the 3- and 9-month follow-ups were collected from patients on a 3-month follow-up schedule only; data at 6 and 12 months were collected from all patients.

^b^VSQ-9: Visit-Specific Questionnaire-9.

^c^HRQoL: health-related quality of life.

Patients stayed in the study for 12 months. All patients completed baseline questionnaires recording sociodemographics (postcode, sex, age, ethnicity, and employment status), time elapsed since transplant, VSQ-9 scores, HRQoL (using EQ-5D-5L), health care use in the previous 3 months, and costs (travel or personal expenses) associated with their baseline appointment. Patients on a 3-month follow-up schedule received questionnaires via myhealth@QEHB [26] up to 7 days after their 3-, 6-, 9-, and 12-month appointments. Patients seen every 6 months received questionnaires at 6 and 12 months. The 6- and 12-month questionnaires collected data on VSQ-9, HRQoL, costs, IT issues, and health service use. The 3- and 9-month questionnaires covered VSQ-9 and costs only. A short questionnaire was also sent to eligible patients who chose not to participate, to understand their decision.

The qualitative interviews covered patients’ experiences of the study (both arms) and recommendations for improvement (intervention arm). Staff interviews focused on experiences of remote consultations and perceived advantages and disadvantages.

### Data Analysis

An intention-to-treat analysis was performed using SPSS version 25 (IBM Corporation) [31]. Participant characteristics at baseline were summarized descriptively and compared between study arms using two-tailed *t* tests or chi-square tests, as appropriate. Analysis of the primary outcome was undertaken using analysis of covariance tests to compare intervention and usual care group satisfaction scores at the study end while controlling for baseline scores. For patients with missing 12-month VSQ-9 data, their most recently available data were used. Secondary outcomes were analyzed descriptively, and feasibility outcomes were presented overall and by group with counts and percentages. Subgroup analyses were not undertaken because of small participant numbers.

For the qualitative process evaluation, interviews were audio-recorded and transcribed verbatim. Transcripts were uploaded to NVivo 12 Plus (QSR International) [32], and with no a priori expectations of findings, the interviews were analyzed thematically [33]. The analysis followed the Braun and Clarke [33] recommended six-stage process of analysis: (1) the researcher spending time familiarizing themselves with the data; (2) the researcher generating initial codes for the data; (3) the researcher starting to develop themes from the codes; (4) the researcher reviewing the themes and codes; (5) the researcher defining, refining, and naming the themes; and (6) the researcher producing an analytic narrative of the findings. Data coding was undertaken by 1 researcher, with 10% of the transcripts independently coded by a second researcher. An initial coding framework was developed using the first 10% of transcripts. If data did not fit the codes in this framework, they were discussed within the team, and where appropriate, new codes were generated, or amendments were made until all data were analyzed. Findings from both patient and staff data sets were compared to identify any similarities or differences.

### Sample Size

The protocol [22] outlined a definitive trial in which a required sample size of 90 patients in each arm (180 total) would be sufficient to detect a 10-point difference in VSQ-9 scores between groups at 80% power and α=.05. This was based on the estimated annual figures of 267 clinically stable liver transplant patients attending routine follow-up in the clinics of interest 1 to 5 years posttransplant, an estimated 60% participation rate, and 30% attrition [34]. However, the actual number of eligible patients, as well as the recruitment rate, were substantially lower than expected, and the decision was made to formally change the study design to a feasibility RCT in which as many participants as possible would be recruited over 4 months without a formal sample size target [35].

### Ethical Approval

This study received a favorable ethical opinion from the West Midlands Solihull Research Ethics Committee on October 24, 2017 (reference: 17/WM/0338). Research governance approval was obtained from the University Hospitals Birmingham NHS Foundation Trust in February 2018 (reference: RRK6080). The study was sponsored by the University of Birmingham.

### Patient and Public Involvement

The patient and public involvement group of the National Institute for Health Research Collaboration for Leadership in Applied Health Research and Care West Midlands (NIHR CLAHRC WM) provided advice on the study design, data collection tools, and outcome selection. A patient representative sat on the study steering committee.

## Results

### Recruitment, Retention, and Crossover

Recruitment took place between March 12, 2018, and July 19, 2018. Overall, 203 patients were potentially eligible according to age and time elapsed since transplant (Multimedia Appendix 4). After further screening, 63.1% (128/203) patients were considered eligible. Of the 128 eligible patients, 72 (56.3%) were not recruited because of the following reasons: 57% (41/72) were not seen by the research team after their appointment, and 43% (31/72) declined participation. Of the 31 decliners, 15 (48%) decliners subsequently returned a questionnaire explaining their decision: 47% (7/15) liked attending hospital or lived nearby, 27% (4/15) lacked appropriate computer equipment or access to myhealth@QEHB [25], 20% (3/15) felt that it would be too difficult to obtain test results or medication locally, and 7% (1/15) disliked the idea of remote consultations.

Of the 203 patients, 56 (27.6%) were recruited. Of the 56 recruited patients, 29 (52%) were allocated to the intervention, and 27 (48%) were allocated to usual care. Approximately 7% (2/27) of usual care patients withdrew after randomization, giving an adjusted recruitment rate of 26.6% (54/203). All remaining patients continued in the study until it closed. Crossover was substantial, with 45% (13/29) of patients changing to usual care: 69% (9/13) because of patient request; 15% (2/13) for health reasons, and 15% (2/13) were changed by the consultant after missed remote appointment or appointments. No harm or adverse events were reported during the study.

### Participant Characteristics

Mean participant age was 48.9 (SD 13.8) years, and 59% (32/54) of patients were men (Table 2). Approximately 37% (20/54) of patients had follow-up appointments every 3 months. Patients in the intervention arm were slightly younger than those receiving usual care, and a greater proportion was men, although none of the baseline characteristics showed a statistically significant difference between groups when means or proportions were assessed. The mean distance between the hospital and participants’ home postcode was 75.3 (range 7-209) miles for all patients—79.6 (range 7-209) miles for intervention patients and 70.3 (range 8-145) miles for usual care patients.

**Table 2:** Baseline characteristics by the study arm (N=54).

Characteristics		Intervention (n=29)	Usual care (n=25)	Overall	Comparison							
					*t* test^a^ (*df*)		Chi-square (*df*)			*P* value		
**Age (years)**					1.78 (52)		N/A^b^			.08		
	Value, mean (SD)	45.8 (14.1)	52.4 (12.9)	48.9 (13.8)								
	Value, median (IQR)	35.0 (35-57.5)	53.0 (43-62.5)	50.0 (35-62.0)								
**Sex, n (%)**						N/A		.03 (1)			.86	
	Male	18 (62)	14 (56)	32 (59)								
	Female	11 (38)	11 (44)	22 (41)								
**Follow-up, n (%)**						N/A		.02 (1)			.89	
	3 months	10 (35)	10 (40)	20 (37)								
	6 months	19 (65)	15 (60)	34 (63)								
**Distance from hospital (miles)**						0.72 (52)		N/A			.47	
	Value, mean (range)	79.6 (7-209)	70.3 (8-145)	75.3 (7-209)								
**Employment, n (%)**						N/A		1.05 (3)			.79	
	Full time	13 (45)	11 (44)	24 (44)								
	Part time	4 (14)	5 (20)	9 (17)								
	Retired	9 (31)	8 (32)	17 (32)								
	Unemployed	3 (10)	1 (4)	4 (7)								
**Qualifications, n (%)**						N/A		2.06 (3)			.56	
	School level	19 (66)	16 (64)	35 (65)								
	Degree level	6 (21)	5 (20)	11 (20)								
	Postgraduate	3 (10)	1 (4)	4 (7)								
	None	1 (3)	3 (12)	4 (7)								

^a^Two-tailed *t* test.

^b^N/A: not applicable.

### Primary Outcome

**Figure 1:**
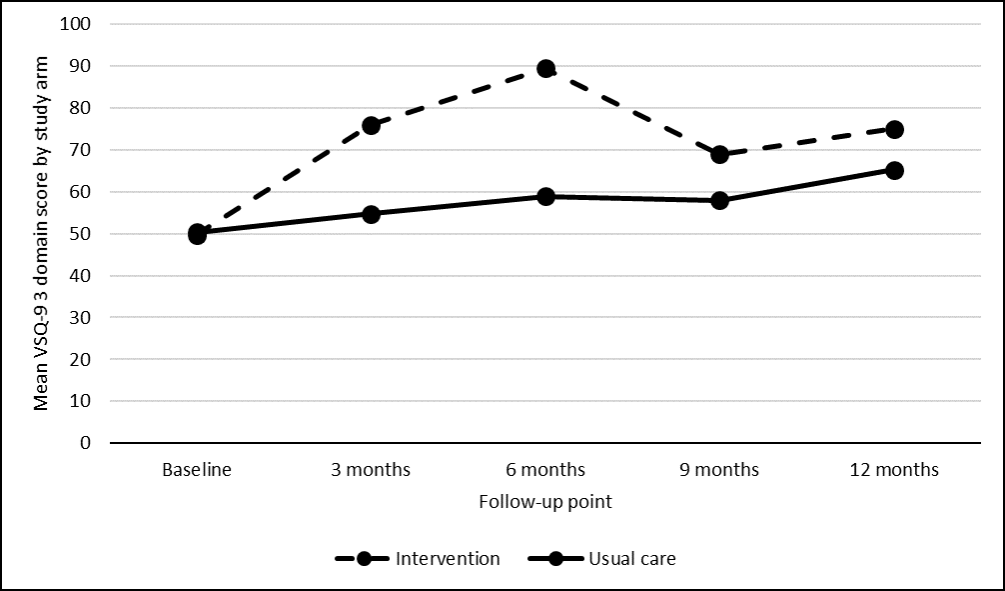
Mean Visit-Specific Questionnaire-9 scores in the three domains comprising the primary outcomes stratified by group. VSQ-9: Visit-Specific Questionnaire-9.

The mean VSQ-9 baseline score for the three domains of *convenience of location*, *getting through by phone*, and *length of time waiting* was 49.7 (SD 17.9) for patients in the intervention arm and 50.3 (SD 19.5) for usual care patients. At each subsequent time point, scores were substantially higher for intervention patients than for usual care patients (Figure 1). At 12 months, scores had increased from baseline in both groups: by 24.7 points for intervention patients (74.4, SD 25.3) and by 14.2 points for usual care (64.5, SD 28.4). Within-group analysis showed that the increase was significant for both intervention (*P*<.001) and usual care (*P*=.02) patients. Analysis of covariance showed no significant difference in satisfaction between groups after controlling for baseline (*F*_1_=2.84; *P*=.10). However, the study was underpowered given its feasibility design.

### Secondary Outcomes

#### Clinical Contacts and System Performance

A total of 129 appointments took place, of which 42 (32.5%) were intended as remote consultations across 19 patients (10 patients had switched to usual care before their first remote appointment). Of the 42 planned remote consultations, 18 (43%) took place over video, and 24 (57%) took place at least partially over the telephone because of technical issues. Only 26% (5/19) of patients received all appointments over video. Approximately 47% (9/19) of patients received a mix of video and telephonic appointments, and the remaining 26% (5/19) received only telephonic appointments. Technical issues reported by questionnaire respondents related to problems with software or browser compatibility, audiovisual issues, system freezes or crashes, log-in problems, and system time-outs.

#### Local Clinical Testing (Intervention Patients)

Blood pressure results were available to the consultant on 81% (34/42) of occasions and weight results on 86% (36/42) of occasions. Blood test results were more variable, with 62% (26/42) of consultations having full blood results, 17% (7/42) having partial results, and 21% (9/42) being without results. The most common reasons for the lack of blood test results were that tests had been done but results not passed on; tests were done, but key analyses were omitted (usually urea, electrolytes, and tacrolimus); the patient did not undergo the tests; or because GPs needed clarification about what was required. These issues decreased over time, with only 1 patient lacking preappointment test results by their 12-month appointment.

#### VSQ-9 Other Domains

The mean VSQ-9 baseline score for the six domains not assessed within the primary outcome (*wait for appointment*, *time with consultant*, *explanation of what was done*, *technical skill of clinician*, *personal manner*, and *overall impression*) was 73.7 (SD 14.0) for intervention patients and 66.9 (SD 14.8) for usual care patients. The scores remained higher for intervention patients at each follow-up point. At the end of the study, satisfaction scores were 80.9 (SD 15.6) for patients receiving the intervention and 72.7 (SD 19.6) for patients receiving usual care.

#### Questionnaire Return and Data Completeness

Approximately 63.6% (82/129) of postappointment questionnaires were returned. A greater proportion of questionnaires was returned by intervention patients: 35.6% (46/129) compared with usual care patients 27.9% (36/129). Approximately 20% (11/54) of patients returned no questionnaires, 30% (16/54) returned one or more questionnaires, and 50% (27/54) returned all questionnaires. Of the 82 questionnaires returned, only 22 (27%) were sent back electronically (11 in each arm); the remainder were submitted on paper. Data on health service use were provided by all intervention and usual care patients, and all questionnaires that were returned at baseline, 6 months, and 12 months from patients in both study arms contained full HRQoL data. Data on patient costs were poorly completed, with fewer than half of all returned questionnaires containing data that could inform an economic evaluation.

### Patient and Staff Experience

Of the 12 interviews conducted for the qualitative process evaluation, 8 (67%) were with patients receiving the intervention and 4 (33%) with staff (2 consultants, 1 member of IT support, and 1 hospital administrator; Table 3). All patient participants preferred their interviews to be conducted over the telephone. All staff interviews were conducted face-to-face on hospital premises. No one else was present at the interviews apart from the participants and researchers. The interview length ranged from 15 to 45 minutes. Six overarching themes were interpreted from the data: (1) the recruitment process; (2) using the myVideoClinic software (including barriers and facilitators); (3) perceptions of remote consultations (including satisfaction with care and views on remote consultations compared with face-to-face appointments); (4) local clinical testing (barriers and facilitators to obtaining tests and ensuring results were made available); (5) perceived benefits of remote consultations to the hospital; and (6) implications for implementation. The findings for each theme are described below, with key verbatim quotations cited in the text. Quotations are attributed using participant number, sex, and age group for patients and using participant number and role for staff. Multimedia Appendix 5 summarizes the key themes (including definitions) and provides supplementary supporting quotations for each theme.

**Table 3:** Characteristics of interview participants (N=12).

Characteristics			Participants, n (%)
**Patients (n=8)**			
	**Sex**		
		Male	4 (50)
		Female	4 (50)
	**Age (years)**		
		30-39	1 (12)
		40-49	2 (25)
		50-59	2 (25)
		60-69	2 (25)
		70-79	1 (12)
**Staff (n=4)**			
	**Sex (n=4)**		
		Male	1 (25)
		Female	3 (75)
	**Information technology (n=1)**		
		Male	0 (0)
		Female	1 (100)
	**Administration (n=1)**		
		Male	0 (0)
		Female	1 (100)
	**Consultant (n=2)**		
		Male	1 (50)
		Female	1 (50)

### Interview Themes

#### The Recruitment Process

Both consultants who were interviewed agreed that appointments in which the study was explained to patients were slightly longer than usual. However, recruitment was not onerous for clinical and administrative staff, and patients were generally keen to participate:

So broadly, it was a straight forward study in some respects to recruit for, because it’s not a particularly evasive intervention for patients. But of course recruiting people in a busy clinical setting is always hard remembering and trying to make sure that you pick the right people.

Participant 02, consultant

However, both consultants also reported that time constraints meant that not all clinic lists could be prescreened to identify potentially eligible patients before appointments began, which affected recruitment rates.

#### Using myVideoClinic Software

Audio and visual problems with at least one remote consultation were reported by 75% (6/8) of patients, and it was rare that sound and video worked together, even when preappointment device testing showed no incompatibility between patients’ home system and the Trust. Connections were often slow, and 1 participant was particularly concerned about the confidentiality of their appointment:

If you’ve got something very private or something you’re really worried about you’d not be very comfortable if the consultant is going “I can’t hear you,” “I can’t hear you.”

Participant 08, female, aged 60-69 years

Both consultants also experienced problems, and many planned video appointments became telephone consultations. IT support was not always timely because of pressure on clinic time:

It was always after the appointment had failed then you’d contact the help desk and they kind of try and work out what had happened at the time which is never quite so good because they can’t see what the problem is.

Participant 01, consultant

All the patients (8/8, 100%) welcomed the facility to upload questions before their appointment, although none used it, nor did any patients report accessing the audio recording of their appointment.

#### Perceptions of Remote Consultations

All patients (8/8, 100%) reported satisfaction with the received care; however, 63% (5/8) explicitly noted that this might not be satisfactory if they were unwell. For example, 1 patient was worried about being unable to demonstrate physical symptoms to her consultant:

The disadvantage is that I can’t physically show anything on my body, so at one stage I did have these rashes, but doctor couldn’t physically examine me.

Participant 08, female, aged 60-69 years

Of the 8 patients, 6 (75%) thought that their patient-physician relationship remained the same and 2 (25%) reported some awkwardness with remote consultations:

It makes you feel a little bit more distant and I would say that I always do feel more reassured when I see them in person.

Participant 12, male, aged 50-59 years

Consultants reported that appointments tended to be more business-like than face-to-face interactions, with less informal discussions. All patient participants (8/8, 100%) reported saving time and money:

If I use the car, then it’s an 80 mile, 2 hour minimum drive. And some day’s it been a 4 hour drive, and that’s each way.

Participant 05, male, aged 70-79 years

For patients reporting negative health impacts from travel (1/8, 13%), the option of a remote appointment was strongly welcomed:

To Birmingham would take me 4 hours, and 4 hours back.... To be perfectly honest, it made me ill for days.

Participant 06, male, aged 60-69 years

#### Local Clinical Testing

Of the 8 patients, 4 (50%) reported that it was convenient to obtain test results locally, but for others, it was challenging, at least at the start of their involvement in the study. GPs were often unsure which tests were needed and whether they were permitted to order them. Sending results to the hospital required pragmatism: 50% (4/8) of the participants uploaded their results via the myhealth portal, but the consultant could not always see them; the remaining 50% (4/8) relied on their local center (eg, GP) to send results; however, this could be problematic:

I had to chase them up a couple of times. When they did, the results weren’t actually transferred onto myhealth.

Participant 08, female, aged 60-69 years

A lack of blood test results was reported by both consultants as making appointments more difficult, as they may not have had the full set of clinical information required for decision-making about patient treatment:

Well, it makes it more difficult to – so you wouldn’t make a decision about changing medications or adjusting doses without blood test results.... If patients say “oh yes I had my bloods and they told me the results were fine” I think it’s important to know what the results actually were rather than just relying on their recollection that the results were fine.

Participant 01, consultant

However, both consultants agreed that when results were available, remote consultations became more efficient compared with standard appointments:

But actually it’s much better than previous [face-to-face appointments], because obviously normally what happens is I take bloods [in clinic] and then I get the results a few days later. Whereas then we have the result discussed in the [video] clinic.

Participant 02, consultant

#### Benefits to the Hospital

Although a health service cost analysis could not be undertaken for this study, remote consultations have the potential to release clinic spaces for those needing face-to-face consultations. This was seen by both consultants as potentially facilitating cost savings for the Trust:

They [patients] don’t have a nurse that’s weighing them and measuring their blood pressure and taking bloods and sending their bloods to the lab for processing. All that I guess is cost saving.

Participant 01, consultant

Being seen as innovative and making use of available technology was also regarded as important, although the need for appropriate space in the hospital for remote consultations was highlighted as a challenge:

You just need an environment. I mean an office is maybe not the best. You almost need a quiet room with the right computer facilities, you know, and no extra noise and no one coming in or out.

Participant 02, consultant

#### Implications for Implementation

Despite some reservations being expressed about issues such as clinical testing and audio or visual problems, all patients and staff who were interviewed welcomed remote consultations and saw these as becoming crucial for routine follow-up care in the future, as long as patients could exercise choice about their appointment type. Trust support for the long-term implementation of remote consultations was seen as essential. In this study, only consultants undertook remote appointments. If the technology was to be used in other specialties, other members of the clinical team, such as registrars, would need to conduct appointments. Consultants were apprehensive about this and about the adjustments required:

I would find it more difficult to, because then I will be still be retaining responsibility for that patients’ care that the registrar has seen in a virtual clinic and although we’re all relatively used to the concept of taking responsibility for patient care when they’re seen by somebody other than you in clinic, I guess it’s just something new that I might get used to.

Participant 01, consultant

However, both consultants considered it prudent to train staff to highlight the differences between face-to-face and remote consultations. With regard to future appointments, 63% (5/8) of patients expressed a clear preference to continue with remote appointments, with the option of attending hospital if necessary:

Maybe I think alternate it, so one on the virtual and another one.... Sort of physically.

Participant 08, female, aged 60-69 years

In contrast, the remaining 38% (3/8) of patients reported that face-to-face appointments would still be their preference. Both consultants felt that remote consultations would be increasingly required, given the rising demand for hospital services while acknowledging that they may not suit all patients:

So I think we have to do something to try and mitigate against that and it’s an obvious solution to some of those issues and I think it will work really well for a number of patients. It won’t work for everybody but I think it would substantially reduce or potentially reduce the number of patients that actually have to come to [the hospital].

Participant 01, consultant

## Discussion

### Principal Findings

Recruitment to our study was lower than expected: the pool of eligible patients was comparatively small, and the recruitment rate was only 26.6% (54/203). Consequently, the study design was altered from a planned definitive trial to one that focused on the feasibility of administering the intervention and collecting evaluative data. Although no participants left the study, the crossover rate from intervention to usual care was high at 45% (12/29), with most changes to the study arm made at the request of the patient before their first remote appointment. This impaired our ability to fully assess processes and outcomes for patients in the intervention group and implies that future evaluations of outpatient remote consultations may benefit from a cohort rather than a randomized design.

### Limitations

The principal limitation of this study was the need to change design from the definitive trial outlined in the published protocol [22] to a feasibility RCT following difficulties with recruitment. Many patients were ineligible because of clinical or technological issues, and recruitment rates were low, with only 54 patients recruited in total. There was a substantial crossover from intervention to usual care, leaving only 30% (16/54) of patients receiving the intervention. Consequently, the study was unable to definitively evaluate the impact of remote consultations on patient satisfaction, and the incidence of technical issues that affected the mode or quality of remote consultations was self-reported and not objectively measured. Similarly, although the baseline characteristics of patients in each study arm were comparable, we could not compare the characteristics of patients recruited to the study with those of the wider liver transplant patient cohort at QEHB, which affects the external validity of our findings. However, our in-depth qualitative process evaluation and assessment of the feasibility of implementing remote consultations for routine follow-up care in the hospital setting may offer important insights to others attempting to establish similar services.

### Comparison With Prior Work

Many existing studies in this field have been undertaken in primary care rather than secondary care, and there are comparatively fewer randomized studies in which a remote consultation intervention is compared with standard or usual care or another appropriate control group. Most importantly, a few studies have assessed some of the practical challenges and facilitators affecting implementation in clinical practice (particularly the views of health care professionals on the feasibility of remote consultations), and to our knowledge, only one other study has assessed remote consultations for recipients of liver transplants [8]. Thus, we believe that our study adds to what is already known on this topic and can provide useful insights for practitioners seeking to implement remote consultations in routine care following their widespread use during the COVID-19 response.

Our overall findings suggest that remote consultations were effective, and despite technical challenges, interview data suggested enthusiasm for care to be delivered remotely in the future. The consultants involved in administering the intervention were similarly enthusiastic for the option of remote consultations to be available in routine practice. As reported by others [9,36], patients in both study groups were satisfied with their care, although the between-group difference was not significant after controlling for baseline scores. However, the substantial increase of 27 points between baseline and study end for patients receiving remote consultations may indicate that appointments undertaken *at a distance* can replace traditional face-to-face consultations without impairing patient satisfaction. This is particularly pertinent with regard to satisfaction scores on the other six domains of VSQ-9—scores for intervention patients remained higher than those in the usual care group even for features of the consultation that may be adversely affected by remote appointments, such as satisfaction with the personal manner of the consultant and perceptions of their technical skills. Maintenance of positive patient-practitioner interactions may have been aided by relationships already having been established in previous face-to-face appointments [37,38]. This may indicate a key advantage of using remote consultations to deliver routine follow-up care within the hospital setting.

Consultants delivering remote consultations reported that the duration of remote consultations was typically shorter than that of face-to-face appointments and more business-like, with small talk kept to a minimum [16,39]. We were unable to assess health service costs; however, it may be reasonable to assume that remote consultations have the potential to save resources for the NHS. It is important to ensure that remote consultations do not simply displace costs associated with routine patient follow-up to other parts of the health and social care system [39]. Our study demonstrated the feasibility of collecting data on health service use, and this should be an important element of future research assessing the service and cost implications of offering remote consultations, particularly when clinical test results must be obtained for review during remote appointments. Obtaining blood test results proved to be challenging for many participants. Although workarounds were found, it is clear that careful consideration of viable processes for local clinical testing is required to make this less burdensome for patients. It did not prove feasible to collect meaningful data on patient costs; however, qualitative evidence showed that remote consultations were perceived to offer cost and time savings for many participants. The opportunity for remote consultation was also beneficial for patients who lived far from the hospital and for whom the journey was considered detrimental to their health [40].

### Implications for Implementation in Clinical Practice

Given the growing demand for hospital services and the increasing pressure on hospital infrastructure, it is likely that more intensive use of remote consultations will be needed in the future to deliver routine patient follow-up care. Our study highlights a number of areas that require consideration to support effective implementation, and our findings also give important insights into whether remote consultations remain acceptable to patients and health care professionals beyond their unprecedented expansion during the COVID-19 response. First, for conditions where the results from clinical tests must be available during consultations, a robust system for patients to obtain these tests locally should be put in place [3,4]. Second, as has been reported by others, technical issues with the myVideoClinic software were frequently experienced by both consultants and patients [9,16], and less than half of remote consultations took place using video as planned. Technology is constantly evolving, and the facility (currently being developed) to offer access to remote consultations via smartphones is likely to resolve many of these technical issues. Other studies have also reported reluctance to use remote consultations on the part of consultants [38], and training should be offered to support the implementation of virtual models of care and address any associated ethical challenges [37]. There may be a core group of patients who do not wish to have remote consultations under any circumstances. Our survey of nonparticipants showed reluctance to have a remote appointment for a range of reasons, including dislike of the idea of remote follow-up, poor engagement with technology, preference for attending in-person because they lived nearby or enjoyed coming to the hospital, or a perception that obtaining medication and clinical tests locally would be too challenging. This suggests that remote consultations should be offered subject to patient choice and support traditional models of follow-up care rather than replacing them [12]. Unanswered questions remain regarding the equity of access to virtual consultations (particularly for rural patients who may not only live far from hospital sites but also experience poor technological infrastructure) and their suitability for diverse clinical specialties in which face-to-face consultation may continue to be necessary.

### Conclusions

The NHS faces substantial pressure on resources and infrastructure because of increasing patient demand. Using technology to support routine patient care may ease some of these pressures. This study demonstrates that using remote consultations in routine follow-up for liver transplant patients is not detrimental to patients’ overall care experience and may have a positive impact on patient satisfaction. During the COVID-19 pandemic, telephone and video-based appointments, rather than face-to-face contacts, have been offered almost exclusively by many NHS Trusts to slow down virus transmission and reduce the strain on NHS services. Although patients and practitioners may have accepted these changes as a temporary measure, our study shows that a number of technical and process issues must be resolved if the routine use of remote consultations is to be acceptable to patients and staff in the postpandemic NHS.

## Appendix

Multimedia Appendix 1 CONSORT-eHEALTH checklist (V1.6.1).

Multimedia Appendix 2 COREQ (Consolidated Criteria for Reporting Qualitative Studies) 32-item checklist.

Multimedia Appendix 3 Topic guide for patient and staff interviews.

Multimedia Appendix 4 Participant flow diagram.

Multimedia Appendix 5 Summary of themes and supplementary quotations from the qualitative process evaluation.

## Abbreviations

CLAHRC: Collaboration for Leadership in Applied Health Research and Care
CONSORT: Consolidated Standards of Reporting Trials
COREQ: Consolidated Criteria for Reporting Qualitative Studies
GP: general practitioner
HRQoL: health-related quality of life
IT: information technology
NHS: National Health Service
NIHR: National Institute for Health Research
QEHB: Queen Elizabeth Hospital Birmingham
RCT: randomized controlled trial
VSQ-9: Visit-Specific Questionnaire-9
WM: West Midlands
