# Supplementary material for: Remote Consultations Versus Standard Face-to-Face Appointments for Liver Transplant Patients in Routine Hospital Care: Feasibility Randomized Controlled Trial of myVideoClinic
Source: J Med Internet Res. 2021 Sep 17;23(9):e19232. doi: 10.2196/19232 (PMC8486986; doi:10.2196/19232)
Supplement: Multimedia Appendix 3 [file jmir_v23i9e19232_app3.doc]

**Multimedia Appendix 3: Topic guide for patients and staff**

The topic guide will be customised as necessary to ensure that the most relevant questions are asked to the appropriate staff and patient groups. The topic guide will be updated to explore emerging issues as more interviews are completed but the topics expected to be covered are listed below.

1. **Patients receiving the intervention**

***Usability of virtual clinic***

- How many virtual clinics did you have?
- What information did you receive?
- What did you think of the information you was given regarding the virtual clinics?
- How did you find the process of doing blood tests locally?
  - Prompts
    - Ease of making an appointment for blood tests
    - Ease of getting the results available on MyHealth prior to the virtual clinic
    - Were blood tests available to the clinician
- Any problems using the virtual clinic?
- Anything that helped when using the virtual clinic?
- Any technical issues?
- Was the system easy to use?
- Was there any reason you did not use the virtual clinic?

***Experience of virtual clinic***

- Please could you tell me about some of your experiences of the using the virtual clinic?
  - Prompts
    - Any benefits?
    - Any disadvantages?
- Is there any way the virtual clinic could be improved?
- What did you think about being able to see the waiting time?
- What did you think about having access to the audio recording of the consultation?
  - Did they use it? What did they think about it?
- Did you find it useful submitting 3 questions prior to your consultation?

***Relationship with medical team***

- Which members of your medical team have you had contact with?
- Has the virtual clinic had an impact on any of your relationships with your medical team?
- How have you found communication between you and your clinical team?

***Satisfaction with care***

- How did you find the appointment process?
- Has the virtual clinic had any impact on your time?
  - Prompts
    - How did waiting times compare to normal face to face appointments?
    - Have you had to take time off work?
- Has the virtual clinic had any impact of your costs?
  - Prompts
    - Were there any different costs associated with the virtual clinic compared to face to face appointments? Parking, driving?
- Did you feel you received all the care and information you needed?
- Was anything missing?
- Was the virtual clinic convenient?
- Was the virtual clinic efficient?
- Are you satisfied with the care you have received?
- Was there any limitations regarding your care?
- Did you feel confident and able to talk to your consultant using the virtual clinic?
  - Did technology get in the way?
- Do you think virtual clinics could also be suitable for earlier consultations?

***MyHealth***

Did the virtual clinic prompt you to use any other aspects of myhealth?

***Preference***

- - Do you have a preference regarding the format of your follow up appointments?
  - Is there anything else you would like to add that I have not covered

1. **Healthcare professionals involved in the care of intervention patient group**

- What do you think of virtual clinics?
- Are there any benefits of using a virtual clinic? (e.g. clinical or person centred)
- What there any disadvantages/issues with using the virtual clinics?
- Are there any strategies in place to overcome these issues?
- Are there any barriers to using virtual clinics in practice? (e.g. costs, time, clinical resistance, targets, commissioning )
- Was data on blood tests available?
- What is needed for this service to run smoothly in practice?
- Are there any key individuals who will make it work?
- Are virtual clinics sustainable?
- Do you perceive any issues with using them in practice? E.g. health professional/patient acceptability, remote testing
- If virtual clinics were used in practice what would need to be done beforehand? (safety issues, staff training, organisational issues, IT issues)
- Anything else to add regarding virtual clinics
