# Supplementary material for: Remote Consultations Versus Standard Face-to-Face Appointments for Liver Transplant Patients in Routine Hospital Care: Feasibility Randomized Controlled Trial of myVideoClinic
Source: J Med Internet Res. 2021 Sep 17;23(9):e19232. doi: 10.2196/19232 (PMC8486986; doi:10.2196/19232)
Supplement: Multimedia Appendix 4 [file jmir_v23i9e19232_app4.doc]

Potentially eligible

n=203

12 month follow-up

n=25

Eligible

n=128

Not recruited n=72

- Not seen by researchers (n=41)
- Declined (n=31)

Not eligible n=75

- Health reasons (n=26)
- Computer equipment (n=26)
- DNA appointment (n=16)
- No local testing (n=3)
- Not fluent in English (n=2)
- Deemed unsuitable (n=1)
- Not on 3/6 month follow-up (n=1)

Withdrew after randomisation

n=2

Consented and randomised

n=56

12 month follow-up

n=29

Allocated to intervention

n=29

Allocated to control

n=27
