# Supplementary material for: Remote Consultations Versus Standard Face-to-Face Appointments for Liver Transplant Patients in Routine Hospital Care: Feasibility Randomized Controlled Trial of myVideoClinic
Source: J Med Internet Res. 2021 Sep 17;23(9):e19232. doi: 10.2196/19232 (PMC8486986; doi:10.2196/19232)
Supplement: Multimedia Appendix 5 [file jmir_v23i9e19232_app5.doc]

**Multimedia appendix 5 – Summary of themes and supplementary supporting quotations from qualitative process evaluation**

| **Theme** | **Definition** | **Supplementary quotations** |
| --- | --- | --- |
| The recruitment process | Reflections on the process and time taken to recruit patients to the study | *“It’s setting aside time to pre-screen in a busy clinic, is not straight forward. But pre-screening I think is key”.* (Participant 02, Consultant) |
| Using the myVideoClinic software | Patient and staff experiences of using the myVideoClinic software and any barriers and facilitators they encountered | *“I guess the difficulty was when I could see a patient was there and waiting but the call wouldn’t connect. So I would then usually speak to the patient on the phone”.* (Participant 01, Consultant) |
|  |  | *“I know a lot of people used the function to put down notes themselves, but they didn’t share them with me”.* (Participant 02, Consultant) |
|  |  | *“I tested it but I never used it. But it works”.* (Participant 03, Female, 50-59) |
| Perceptions of remote consultations | Participants’ thoughts about remote consultations. Their satisfaction with the care they received and their views on their remote consultation compared to a face-to-face appointment | *“I think in a way it makes the appointment a bit more business-like, because they want to get on with their day and with them being at home, there’s no need to kind of bump it out a bit longer than normal”.* (Participant 02, Consultant) |
|  |  | *“Well, it’s amazing, we usually have to pay for dog minders”.* (Participant 07, Female, 40-49) |
| Local clinical testing | Barriers and facilitators to obtaining tests (i.e. blood tests) locally and ensuring that the results were available to the consultant at the time of the appointment. | *“I just emailed them there and then to [consultant] email address and [consultant] was able to go through it with me there and then”.* (Participant 07, Female, 40-49) |
| Benefits to the hospital | Participants views on how the use of remote consultations for routine hospital follow-up appointments can be beneficial to the hospital | *“I guess there are also advantages in being kind of seen as, ……… keeping up with modern technology and trying to incorporate modern technology into how we look after patients and that’s something that’s probably important to engage our patients in the longer term”.* (Participant 01, Consultant) |
| Implications for implementation | The perceived barriers and facilitators to a wider long-term rollout of remote consultations. | *“I have the impression that they are keen to pursue it, so I would hope they would support things to enable that to happen”.* (Participant 01, Consultant) |
|  |  | *“I guess just to raise awareness of the potential risks involved in doing the video clinic as opposed to doing a face-to-face clinic. In face-to-face clinics there are several safety nets in place that we don’t even think about. I think some traditional training probably would be required just to raise peoples’ awareness”.* (Participant 01, Consultant) |
|  |  | *“Even though the doctor will be on the other side of the screen I still would rather be there with them”.* (Participant 03, Female, 30-40) |
